# U-shaped relationship between fasting blood glucose and urinary albumin-to-creatinine ratio in the general United States population

**DOI:** 10.3389/fendo.2024.1334949

**Published:** 2024-03-15

**Authors:** Jianling Song, Ping Wang, Hong Li

**Affiliations:** ^1^ Department of Nephrology, The Second Affiliated Hospital, Jiangxi Medical College, Nanchang University, Nanchang, Jiangxi, China; ^2^ Department of Gynecology and Obstetrics, Yongfeng People’s Hospital, Jian, Jiangxi, China; ^3^ Department of Medical Records, The Second Affiliated Hospital, Jiangxi Medical College, Nanchang University, Nanchang, Jiangxi, China

**Keywords:** fasting blood glucose, urinary albumin-to-creatinine ratio, albuminuria, NHANES, U-shaped relationship

## Abstract

**Objective:**

The current controversy surrounding the association between fasting blood glucose (FBG) and albuminuria necessitates further investigation. Hence, the primary objective of this study was to examine the relationship between FBG and urinary albumin-to-creatinine ratio (UACR).

**Methods:**

A cohort of complete data from National Health and Nutrition Examination Survey (NHANES) participants (1999-2020) was analyzed. Linear regression analyses and a generalized additive model explored the association between FBG and UACR. Furthermore, the stability of this relationship across different populations was assessed.

**Results:**

The study involved a total of 20,264 participants who were identified as U.S. citizens. By employing linear regression analysis, a statistically significant relationship was observed between elevated FBG levels and an increase in UACR (P<0.0001). Additionally, using a generalized additive model analysis, a U-shaped correlation between FBG and UACR was identified. Further examination using threshold effect analysis indicated a turning point for FBG at 5.44 mmol/L. A noteworthy finding in multiple populations is the consistent U-shaped association between FBG and UACR, except for individuals with serum uric acid levels ≥420 μmol/L and those who refrain from alcohol consumption.

**Conclusion:**

The general U.S. population has a U-shaped nonlinear relationship between FBG and UACR.

## 1 Introduction

Microalbuminuria indicates the initial stages of glomerular disease and poses a risk for the progression of macroalbuminuria (1, 2). Numerous studies have demonstrated a correlation between substantial albuminuria, an elevated likelihood of developing end-stage renal disease, and an increased mortality rate due to coronary heart disease (3–6). It has been well-established that maintaining blood pressure within optimal ranges effectively reduces albuminuria (7, 8). Furthermore, empirical evidence supports the efficacy of glucose-lowering medications, such as empagliflozin and sitagliptin, in ameliorating albuminuria (8–10).

The relationship between fasting blood glucose (FBG) and albuminuria has generated conflicting findings in the literature. Previous studies have suggested that poor glycemic control is a risk factor for microalbuminuria, which contributes to macroalbuminuria development (11, 12). For instance, Yimthiang et al. observed a 5-fold increase in the odds of developing albuminuria when blood glucose levels exceeded 180 mg/dL (13). However, a separate study conducted among South Asian patients with type 2 diabetes did not find a significant association between a decrease in FBG and a decrease in albuminuria (14). The study by Lei B. et al. (15) did not provide conclusive evidence regarding the potential benefit of reducing blood glucose levels on the development of albuminuria. Furthermore, there is ongoing debate regarding the effectiveness of intensive glycemic therapy in improving albuminuria. However, a separate study indicated that intensive blood glucose therapy was associated with a decreased risk of both microalbuminuria and macroalbuminuria (16). However, it should be emphasized that intensive glycemic control did not yield a statistically significant decrease in the advancement of clinical albuminuria. Furthermore, the longitudinal treatment effect of both conventional and intensive glycemic therapy did not demonstrate statistical significance compared to the rate of clinical albuminuria progression (17). It is worth noting that the significant challenge posed by intensive glycemic therapy includes the risk of hypoglycemia (18), weight gain, and an increased prevalence of metabolic syndrome (19). Hence, given the inconclusive correlation between blood glucose and albuminuria and the uncertainty surrounding the efficacy of intensive glucose treatment in ameliorating albuminuria, it becomes imperative to ascertain a precise glucose threshold. This study sought to examine the association between FBG levels and albuminuria to establish a definitive blood glucose threshold, thereby furnishing valuable insights to bolster clinical glucose control therapy.

## 2 Methods

### 2.1 Study population

NHANES, which stands for National Health and Nutrition Examination Survey, is a nationally representative survey conducted by the Centers for Disease Control and Prevention (CDC) in the U.S. The survey utilizes a complex sampling design to ensure that the participants are representative of the entire U.S. population in terms of age, gender, race, ethnicity, income, and education levels.

The Institutional Review Board (IRB) of the National Center for Health Statistics (NCHS) has approved the study methods of NHANES for health statistics. The study methods include collecting data about the participants’ health and nutritional status through interviews, physical examinations, and laboratory tests. This data is used to monitor the prevalence of various diseases and health conditions in the U.S. population, as well as to identify risk factors and trends over time.

To participate in NHANES, volunteers are selected randomly and invited to participate. They are provided with detailed information about the survey and are asked to provide written informed consent before participating (20). The privacy and confidentiality of the participants are strictly protected throughout the survey process. Volunteers who participate in NHANES are contributing to a better understanding of the health and nutritional status of the U.S. population, which can inform public health policies and interventions.

**Figure 1:**
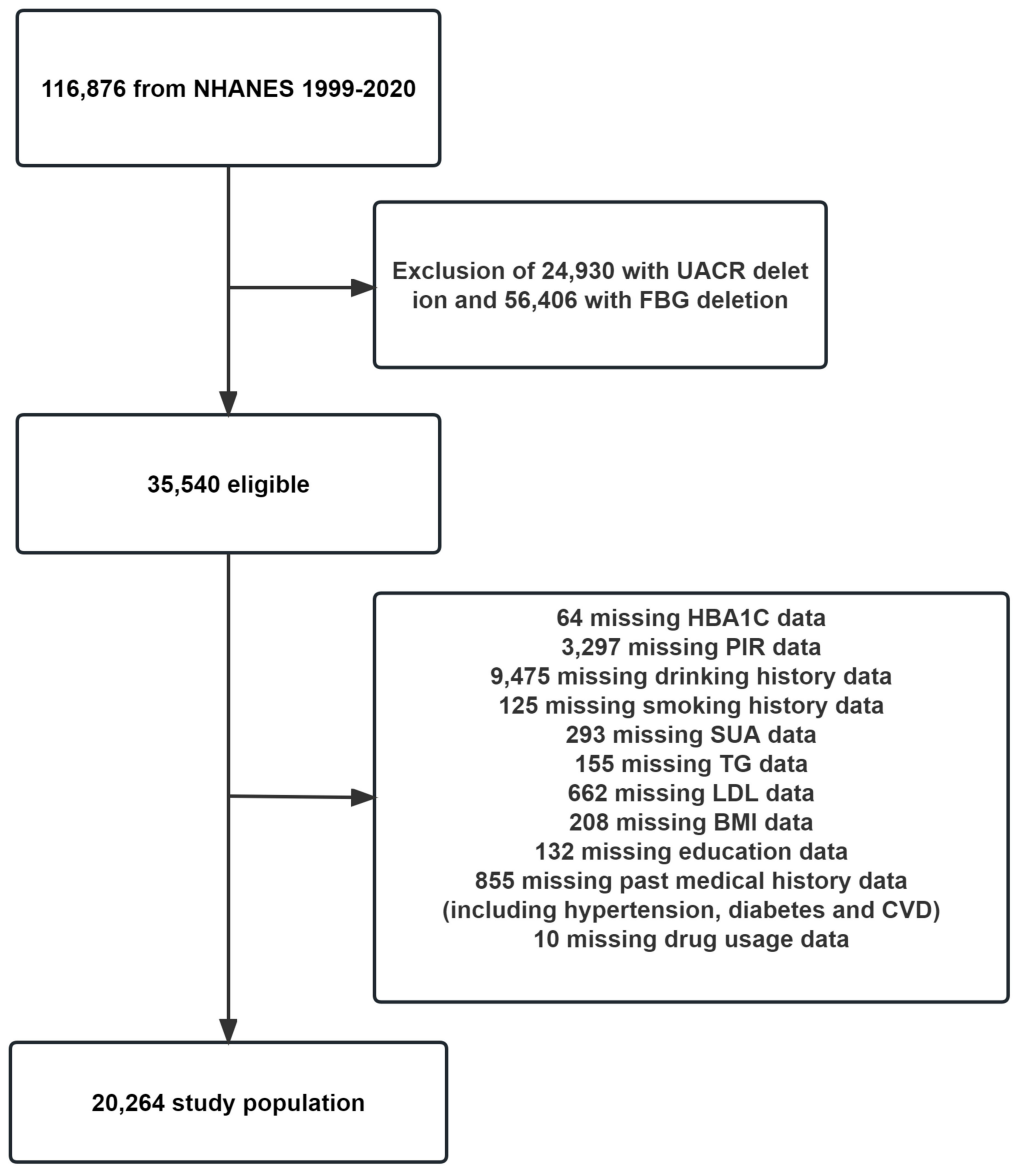
Flowchart for inclusion of study participants. PIR, poverty income ratio; FBG, fasting blood glucose; HbA1C, hemoglobin A1c; SUA, serum uric acid; TG, triglyceride; TC, total cholesterol; LDL, low-density lipoprotein; UACR, urinary albumin-to-creatinine ratio; BMI, body mass index; CVD, cardiovascular disease.

This study focused on a group of U.S. citizens who participated in the NHANES between 1999 and 2020. The survey included 116,876 participants. To ensure the accuracy of our findings, incomplete data were excluded from our analysis. Specifically, participants who had incomplete data on the independent variable FBG (56,406 individuals), the dependent variable urinary albumin-to-creatinine ratio (UACR) (24,930 individuals), and the covariates [including hemoglobin A1c (HbA1C), poverty income ratio (PIR), serum uric acid (SUA), triglyceride (TG), low-density lipoprotein (LDL), body mass index (BMI), education level, history of tobacco and alcohol use, history of prior illness(including hypertension, diabetes and CVD), and history of taking prescription medications] were excluded from our study, amounting to a total of 15,276 individuals. A total of 20,264 individuals were ultimately incorporated into the study. Please refer to **Figure 1** for a detailed breakdown of the exclusions.

### 2.2 Outcome measurements

The primary indicator of the outcome for this study was the UACR. To determine albumin levels, urine samples were subjected to solid-phase fluorescence immunoassay, while urinary creatinine levels were analyzed using the enzymatic creatinase technique on Roche/Hitachi Modular P chemistry analyzer. The use of these methods ensured accurate and precise measurements of albumin and creatinine levels in the urine samples.

### 2.3 FBG and covariate acquisition

The NHANES dataset provides information on participant demographics such as gender, age, race, education level, and PIR, which were collected through questionnaires. The dataset also includes participants’ hematological indicators, including FBG, HbA1C, TG, total cholesterol (TC), LDL, high-density lipoprotein (HDL), creatinine (Cr), blood urea nitrogen (BUN), and SUA, which were directly obtained from the database. Estimated glomerular filtration rate (eGFR) was calculated using the CKD Epidemiology Collaboration (CKD-EPI) equation (21). BMI was calculated based on height and weight measurements (weight/height squared).

Information on alcohol and tobacco usage was collected through questionnaires as well. Participants were asked about their drinking history in the past 12 months and were defined as non-drinkers if they answered “Never”. Otherwise, they were considered drinkers. Similarly, smoking history was obtained by asking participants if they currently smoke cigarettes. Those who answered “yes” were defined as smokers, while those who answered “no” were considered non-smokers.

The participants’ past medical history was gathered through questionnaires and physical examination. Hypertension was diagnosed by asking if they had high blood pressure or were taking medication for it, or by measuring their blood pressure three times (systolic ≥ 140 mmHg or diastolic ≥ 90 mmHg). Diagnosis of diabetes was obtained by questionnaire (self-reporting that they had diabetes or were currently taking medication for glycemic control), FBG ≥7 mmol/L, or HbA1C ≥6.5%. Cardiovascular disease (CVD) is defined as an affirmative answer to the question: Has a doctor or other health professional ever told you that you have congestive heart failure/coronary heart disease/angina/heart attack/stroke? Chronic kidney disease (CKD) was diagnosed as eGFR <60ml/min/1.73m^2^ or UACR ≥30mg/g (22, 23). It is imperative to note that the data concerning participants’ use of prescription medication was obtained solely through participants’ self-reports.

### 2.4 Statistical analysis

Continuous variables in this study did not conform to normal distribution and are expressed as median (Q1-Q3). Categorical variables are expressed as percentages. Linear regression models were constructed to assess the linear relationship between FBG and UACR. We used three models to assess the magnitude of the independent effect of FBG on UACR. Next, we analyzed the nonlinear relationship between FBG and UACR using a generalized summation model. Again, we used three models, where the first model was not adjusted for any variables. Model I was adjusted for sex, age, race, PIR, and education level, while model II was further adjusted for Cr, SUA, BUN, TG, HDL, LDL, eGFR, BMI, smoking, drinking, diabetes, hypertension, CVD, glucose-lowering therapy, antihypertension therapy, and hypolipemic therapy. We selected the adjusted covariates based on regression coefficients greater than 10% for UACR or p-values less than 0.1 for the regression coefficients of covariates with FBG. Finally, we investigated whether a stable non-linear relationship existed between FBG and UACR by dividing participants into subgroups based on different cut-points.

## 3 Results

### 3.1 Baseline characteristics of study participants by UACR grouping

This study encompassed a cohort of 20,264 U.S. citizens actively engaged in the NHANES from 1999 to 2020. The mean age of the participants included in this analysis was 50.00 (35.00-64.00) years. The gender distribution was approximately equal, with a notable representation of Mexican American individuals. Moreover, a substantial proportion of the participants possessed college or higher educational qualifications. Subsequently, we classified the participants above into three distinct categories based on the Kidney Disease: Improving Global Outcomes (KDIGO) guideline recommendations for the stratification of UACR: <30 mg/g, 30-300 mg/g, and >300 mg/g (23). As indicated in **Table 1**, <30 mg/g, 30-300 mg/g, and >300 mg/g had participant counts of 17,803, 2,063, and 399, respectively. Our analysis revealed that individuals in the >300mg/g group exhibited lower PIR and eGFR levels, as well as higher levels of FBG, HbA1C, Cr, BUN, TG, LDL, UACR, and BMI in comparison to the <30mg/g and 30-300mg/g groups. Furthermore, participants in the >300mg/g group demonstrated a higher prevalence of smoking, hypertension, diabetes, and CVD, along with a more significant proportion of individuals taking prescription medications.

**Table 1:** Demographic information and baseline information on the study population, NHANES 1999-2020.

UACR categorical (mg/g)	Total	<30	30-300	>300	P value
N	20,264	17,803	2,062	399	–
Age (years)	50.00 (35.00-64.00)	48.00 (34.00-62.00)	62.00 (47.00-74.00)	63.00 (52.00-74.00)	<0.001
Sex [N (%)]					0.006
Female	10,116 (49.92%)	8,886 (49.91%)	1,060 (51.41%)	170 (42.61%)	
Male	10,148 (50.08%)	8,917 (50.09%)	1,002 (48.59%)	229 (57.39%)	
Race [N (%)]					<0.001
Non-Hispanic white	3,370 (16.63%)	2,898 (16.28%)	390 (18.91%)	82 (20.55%)	
Non-Hispanic black	4,045 (19.96%)	3,455 (19.41%)	475 (23.04%)	115 (28.82%)	
Mexican American	9,306 (45.92%)	8,290 (46.57%)	895 (43.40%)	121 (30.33%)	
Other	3,543 (17.48%)	3,160 (17.75%)	302 (14.65%)	81 (20.30%)	
Education level [N (%)]					<0.001
Junior high school education or below	4,872 (24.04%)	4,043 (22.71%)	683 (33.12%)	146 (36.59%)	
High school education	4,676 (23.08%)	4,072 (22.87%)	499 (24.20%)	105 (26.32%)	
College education or above	10,716 (52.88%)	9,688 (54.42%)	880 (42.68%)	148 (37.09%)	
PIR (%)	2.62 ± 1.62	2.67 ± 1.63	2.31 ± 1.53	2.10 ± 1.44	<0.001
FBG (mmol/L)	5.55 (5.16-6.11)	5.50 (5.14-6.00)	5.94 (5.33-7.05)	6.50 (5.55-9.66)	<0.001
HbA1C (%)	5.50 (5.20-5.80)	5.50 (5.20-5.80)	5.70 (5.40-6.40)	6.30 (5.60-8.10)	<0.001
Cr (mmol/L)	74.26 (61.90-88.40)	73.37 (61.90-88.40)	76.91 (61.88-95.47)	98.12 (73.81-137.02)	<0.001
SUA (μmol/L)	321.20 (267.70-380.70)	321.20 (267.70-374.70)	333.10 (273.60-398.50)	386.60 (321.20-452.00)	<0.001
BUN (mmol/L)	4.64 (3.57-5.71)	4.64 (3.57-5.71)	5.00 (3.93-6.43)	6.78 (4.64-9.64)	<0.001
TG (mmol/L)	1.16 (0.81-1.71)	1.15 (0.79-1.68)	1.29 (0.90-1.88)	1.40 (0.99-2.02)	<0.001
TC (mmol/L)	4.91 (4.24-5.64)	4.91 (4.24-5.64)	4.89 (4.19-5.64)	4.78 (4.16-5.74)	0.901
HDL (mmol/L)	1.32 (1.09-1.60)	1.32 (1.11-1.60)	1.29 (1.06-1.60)	1.24 (1.06-1.58)	0.019
LDL (mmol/L)	2.90 (2.33-3.54)	2.92 (2.33-3.54)	2.82 (2.25-3.52)	2.79 (2.17-3.54)	0.002
UACR (mg/g)	6.89 (4.44-13.45)	6.15 (4.20-10.00)	56.64 (39.08-95.88)	725.42 (430.76-1603.61)	<0.001
eGFR (mL/min/1.73m^2^)	95.74 (79.34-110.54)	96.95 (81.46-111.23)	86.27 (66.00-104.96)	65.85 (42.14-92.52)	<0.001
BMI (Kg/m^2^)	27.90 (24.30-32.40)	27.76 (24.23-32.20)	28.80 (24.80-34.01)	29.40 (24.63-35.14)	<0.001
Smoking [N (%)]					<0.001
No	10,909 (53.83%)	9,688 (54.42%)	1,036 (50.24%)	185 (46.37%)	
Yes	9,355 (46.17%)	8,115 (45.58%)	1,026 (49.76%)	214 (53.63%)	
Drinking [N (%)]					<0.001
No	2,656 (13.11%)	2,255 (12.67%)	329 (15.96%)	72 (18.05%)	
Yes	17,608 (86.89%)	15,548 (87.33%)	1,733 (84.04%)	327 (81.95%)	
Diabetes [N (%)]					<0.001
No	17,725 (87.47%)	16,079 (90.32%)	1,461 (70.85%)	185 (46.37%)	
Yes	2,539 (12.53%)	1,724 (9.68%)	601 (29.15%)	214 (53.63%)	
Hypertension [N (%)]					<0.001
No	11,822 (58.34%)	11,056 (62.10%)	702 (34.04%)	64 (16.04%)	
Yes	8,442 (41.66%)	6,747 (37.90%)	1,360 (65.96%)	335 (83.96%)	
CVD [N (%)]					<0.001
No	18,095 (89.30%)	16,232 (91.18%)	1,587 (76.96%)	276 (69.17%)	
Yes	2,169 (10.70%)	1,571 (8.82%)	475 (23.04%)	123 (30.83%)	
CKD [N (%)]					<0.001
No	16,753 (82.67%)	16,753 (94.10%)	–	–	
Yes	3,511 (17.33%)	1,050 (5.90%)	2,062 (100.00%)	399 (100.00%)	
Drug use [N (%)]					
Glucose-lowering therapy	463 (2.28%)	323 (1.81%)	108 (5.24%)	32 (8.02%)	<0.001
Antihypertension therapy	3,820 (18.85%)	3,022 (16.97%)	632 (30.65%)	166 (41.60%)	<0.001
Hypolipemic therapy	580 (2.86%)	516 (2.90%)	60 (2.91%)	4 (1.00%)	0.080

Continuous variables are expressed using medians (Q1-Q3) and categorical variables are expressed using percentages.

PIR, poverty income ratio; FBG, fasting blood glucose; HbA1C, hemoglobin A1c; Cr, creatinine; SUA, serum uric acid; BUN, blood urea nitrogen; TG, triglyceride; TC, total cholesterol; HDL, high-density lipoprotein; LDL, low-density lipoprotein; UACR, urinary albumin-to-creatinine ratio; eGFR, estimated glomerular filtration rate; BMI, body mass index; CVD, cardiovascular disease; CKD, chronic kidney disease.

Furthermore, **Supplementary Table 1** presents the factors that exhibit an association with UACR. These factors include age, FBG, HbA1C, Cr, SUA, BUN, TG, BMI, presence of hypertension, diabetes mellitus, CVD, and CKD, all of which demonstrate a positive correlation with elevated UACR. Conversely, being of Mexican American ethnicity, possessing a high school education or higher, having a favorable economic status, engaging in current alcohol consumption, undergoing lipid-lowering therapy, and having an eGFR exhibit a negative association with elevated UACR.

### 3.2 Analysis of linear and nonlinear association between FBG and UACR

In order to assess the magnitude of the independent impact of FBG on UACR, we initially conducted a linear regression analysis (**Table 2**) to examine the linear association between FBG and UACR. Our findings revealed a positive correlation between FBG and UACR. More specifically, after adjusting for various confounding factors, we observed that for each 1 mmol/L rise in FBG, UACR increased by 4.67 mg/g (95% CI: 2.51, 6.83). Furthermore, UACR was classified into three groups utilizing clinical cut-points: <30mg/g, 30-300mg/g, and >300mg/g. Our analysis revealed a consistent association between FBG levels and UACR, indicating that elevated FBG levels led to heightened excretion of UACR.

**Table 2:** Regression analysis of the linear relationship between fasting blood glucose and urinary albumin-to-creatinine ratio.

Exposure	Crude model	Model I	Model II
UACR<30mg/g			
FBG	0.69 (0.64, 0.74) <0.0001	0.56 (0.50, 0.61) <0.0001	0.47 (0.41, 0.54) <0.0001
30≥UACR ≤ 300 mg/g			
FBG	1.79 (0.93, 2.65) <0.0001	1.55 (0.68, 2.42) 0.0005	1.31 (0.27, 2.36) 0.0139
UACR>300 mg/g			
FBG	33.46 (-4.87, 71.80) 0.0879	30.02 (-8.97, 69.01) 0.1321	15.95 (-27.52, 59.42) 0.4724
Total			
FBG	4.70 (2.88, 6.53) <0.0001	5.00 (3.13, 6.87) <0.0001	4.67 (2.51, 6.83) <0.0001

FBG, fasting blood glucose; UACR, urinary albumin-to-creatinine ratio.

Crude model: No variables were adjusted.

Model I: Adjusted for gender, age, race, poverty income ratio, and education level.

Model II: Adjusted for gender, age, race, poverty income ratio, education level, creatinine; serum uric acid; blood urea nitrogen; triglyceride; high-density lipoprotein; low-density lipoprotein; estimated glomerular filtration rate; body mass index; smoking; drinking; diabetes; hypertension; cardiovascular disease; glucose-lowering therapy, antihypertension therapy, hypolipemic therapy.

**Figure 2:**
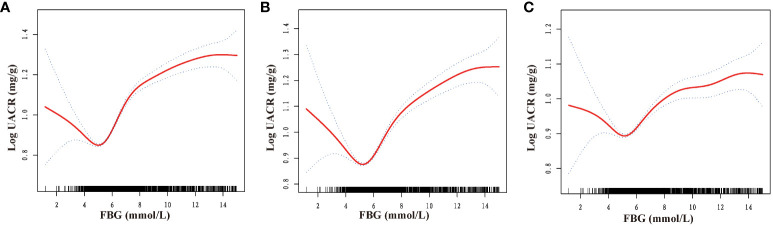
Dose-response relationship between fasting blood glucose and urinary albumin-to-creatinine ratio. FBG, fasting blood glucose; UACR, urinary albumin-to-creatinine ratio. **(A)** No variables were adjusted; **(B)** Adjusted for gender, age, race, poverty income ratio, and education level; **(C)** Adjusted for gender, age, race, poverty income ratio, education level, creatinine; serum uric acid; blood urea nitrogen; triglyceride; high-density lipoprotein; low-density lipoprotein; estimated glomerular filtration rate; body mass index; smoking; drinking; diabetes; hypertension; cardiovascular disease; glucose-lowering therapy, antihypertension therapy, hypolipemic therapy.

Furthermore, a comprehensive examination investigated the nonlinear regression between FBG and UACR. The graphical representation in **Figure 2** clearly illustrates a U-shaped nonlinear association between FBG and UACR. Subsequently, the determination of the inflection point of FBG was accomplished through threshold effect analysis. The data presented in **Table 3** reveals that in the Crude model when FBG is less than 4.69mmol/L, a one mmol/L increase in FBG results in a 210.49 mg/g decrease in UACR. Conversely, when FBG exceeds 4.69 mmol/L, a one mmol/L increase in FBG leads to a 26.15 mg/g increase in UACR. After controlling for gender, age, race, PIR, and education level, the U-shaped association between FBG and UACR remained evident. Specifically, when FBG was less than 4.84 mmol/L, each mmol/L increase in FBG resulted in a 169.66 mg/g decrease in UACR. On the contrary, in cases where FBG exceeded 4.84 mmol/L, a rise of 1 mmol/L in FBG led to a 24.46 mg/g reduction in UACR. Additionally, in the comprehensive adjusted model, it was observed that in cases where FBG levels were below 5.44 mmol/L, a one mmol/L increase in FBG resulted in a 2.17 mg/g decrease in UACR. Conversely, when FBG levels exceeded 5.44 mmol/L, a one mmol/L increase in FBG led to an increase of 2.13 mg/g in UACR.

**Table 3:** Threshold effect of fasting blood glucose on the urinary albumin-to-creatinine ratio analyzed using piece-wise linear regression.

Outcome:	UACR (mg/g)		
	β (95% CI)	P-value	P nonlinear value (P for log-likelihood ratio test)
Crude model			
FBG < 4.69 (mmol/L)	-210.49 (-249.98, -171.01)	<0.0001	<0.001
FBG > 4.69 (mmol/L)	26.15 (23.89, 28.41)	<0.0001	
Model I			
FBG < 4.84 (mmol/L)	-169.66 (-201.80, -137.51)	<0.0001	<0.001
FBG > 4.84 (mmol/L)	24.46 (22.12, 26.80)	<0.0001	
Model II			
FBG < 5.44 (mmol/L)	-2.17 (-3.58, -0.77)	0.0025	<0.001
FBG > 5.44 (mmol/L)	2.13 (1.84, 2.41)	<0.0001	

Crude model: No variables were adjusted.

Model I: Adjusted for gender, age, race, poverty income ratio, and education level.

Model II: Adjusted for gender, age, race, poverty income ratio, education level, creatinine; serum uric acid; blood urea nitrogen; triglyceride; high-density lipoprotein; low-density lipoprotein; estimated glomerular filtration rate; body mass index; smoking; drinking; diabetes; hypertension; cardiovascular disease; glucose-lowering therapy; antihypertension therapy; hypolipemic therapy.

### 3.3 U-shaped relationship between FBG and UACR in different subgroups

**Figure 3:**
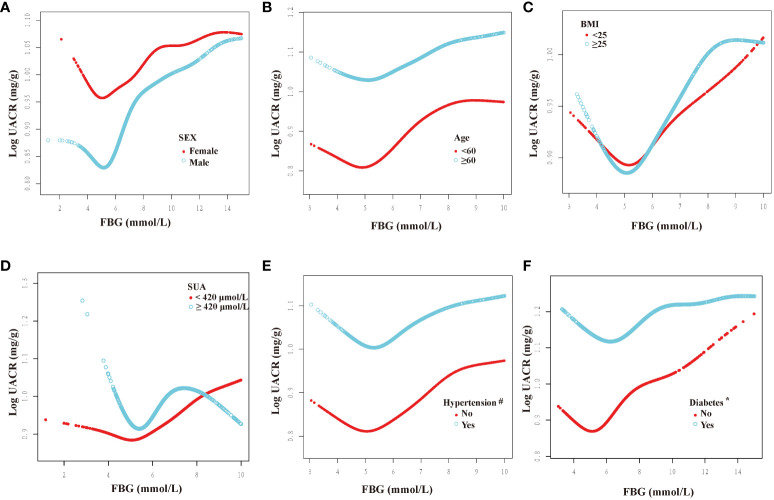
Dose-response relationship between fasting blood glucose and urinary albumin-to-creatinine ratio in different subgroups (I). FBG, fasting blood glucose; UACR, urinary albumin-to-creatinine ratio; SUA, serum uric acid; BMI, body mass index. All subgroups adjusted for gender, age, race, poverty income ratio, education level, creatinine; serum uric acid; blood urea nitrogen; triglyceride; high-density lipoprotein; low-density lipoprotein; estimated glomerular filtration rate; body mass index; smoking; drinking; diabetes; hypertension; cardiovascular disease; glucose-lowering therapy, antihypertension therapy, hypolipemic therapy. The above subgroups were adjusted for the variables, except for themselves. #: This subgroup was adjusted for the variables, except for themselves and antihypertension therapy. *: This subgroup was adjusted for the variables, except for themselves and glucose-lowering therapy. **(A)**: grouped according to sex; **(B)**: grouped according to age; **(C)**: grouped according to BMI; **(D)**: grouped according to the presence of hyperuricemia; **(E)**: grouped according to the presence of hypertension; **(F)**: grouped according to the presence of diabetes.

**Figure 4:**
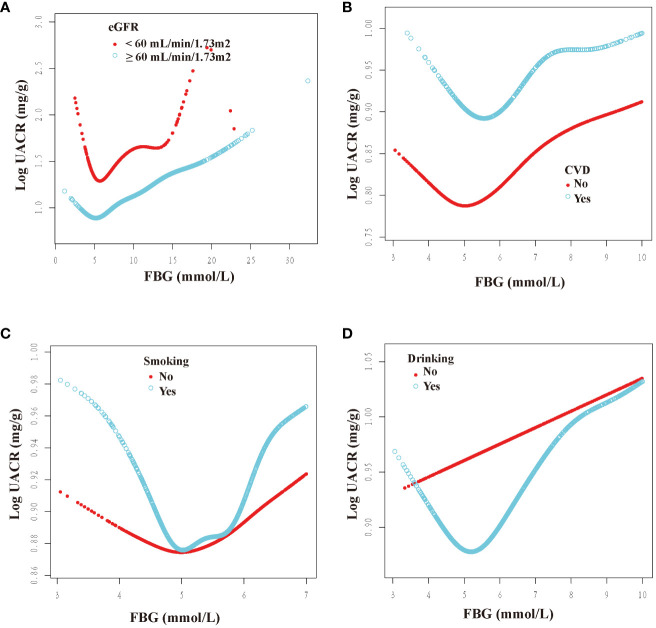
Dose-response relationship between fasting blood glucose and urinary albumin-to-creatinine ratio in different subgroups (II). FBG, fasting blood glucose; UACR, urinary albumin-to-creatinine ratio; CVD, cardiovascular disease; eGFR, estimated glomerular filtration rate. All subgroups adjusted for gender, age, race, poverty income ratio, education level, creatinine; serum uric acid; blood urea nitrogen; triglyceride; high-density lipoprotein; low-density lipoprotein; estimated glomerular filtration rate; body mass index; smoking; drinking; diabetes; hypertension; cardiovascular disease; glucose-lowering therapy, antihypertension therapy, hypolipemic therapy. The above subgroups were adjusted for the variables, except for themselves. **(A)**: grouped according to the presence of renal insufficiency; **(B)**: grouped according to the presence of CVD; **(C)**: grouped according to the presence of smoking; **(D)**: grouped according to the presence of drinking.

We categorized participants based on their demographic characteristics, baseline levels, past medical history, and drinking history in order to examine the consistency of the association between FBG and UACR across different populations. The results presented in **Figures 3**, **4** demonstrate a U-shaped relationship between FBG and UACR in various subgroups, including gender (male/female), age (<60/≥60 years), BMI (<25/≥25 Kg/m^2^), eGFR (<60/≥60 ml/min/1.73m^2^) and SUA <420 μmol/L, as well as in hypertension/non-hypertension, diabetic/non-diabetic, CVD/non-CVD, smokers/non-smokers, and drinkers. However, a U-shaped correlation between FBG and UACR was not observed in the population with SUA levels ≥420 μmol/L and non-drinkers.

Furthermore, inflection points were identified within the subgroups, as presented in **Table 4**. Remarkably, these inflection points exhibited inconsistency across various factors, including gender, age, BMI, SUA, past medical history, and drinking history. Specifically, the inflection point for women was determined to be 5.22 mmol/L, whereas for men, it was 5.61 mmol/L. Additionally, individuals below 60 exhibited an inflection point of 5.38 mmol/L, while those aged 60 and above had an inflection point of 4.83 mmol/L. Furthermore, individuals with an SUA below 420 μmol/L displayed an inflection point of 5.61 mmol/L. The inflection point for individuals with a BMI less than 25 Kg/m^2^ was 5.22 mmol/L, whereas for those with a BMI greater than or equal to 25 Kg/m^2^, the inflection point was 5.61 mmol/L. Furthermore, the inflection point differed between non-hypertension individuals (6.00 mmol/L) and hypertension individuals (5.44 mmol/L), as well as between non-diabetic individuals (5.72 mmol/L) and diabetic individuals (5.11 mmol/L). Additionally, the inflection point for individuals without CVD was 4.96 mmol/L, whereas for those with CVD, it was 5.62 mmol/L. In the population with an eGFR less than 60 ml/min/1.73m^2^, the inflection point was observed at 5.88 mmol/L, whereas in the population with an eGFR greater than or equal to 60 ml/min/1.73m^2^, it was noted at 5.46 mmol/L. Additionally, in the population that consumed alcohol, the inflection point was measured at 5.53 mmol/L.

**Table 4:** Threshold effect of fasting blood glucose on the urinary albumin-to-creatinine ratio analyzed using piece-wise linear regression in different subgroups.

Outcome:	UACR (mg/g)		
	β (95% CI)	P-value	P nonlinear value (P for log-likelihood ratio test)
Female			<0.001
FBG < 5.22 (mmol/L)	-2.26 (-4.63, 0.10)	0.0611	
FBG > 5.22 (mmol/L)	2.06 (1.49, 2.62)	<0.0001	
Male			<0.001
FBG < 5.61 (mmol/L)	-2.14 (-3.95, -0.32)	0.0209	
FBG > 5.61 (mmol/L)	2.46 (1.87, 3.05)	<0.0001	
Age < 60			<0.001
FBG < 5.38 (mmol/L)	-0.71 (-2.12, 0.70)	0.3215	
FBG > 5.38 (mmol/L)	3.10 (2.36, 3.84)	<0.0001	
Age ≥ 60			0.004
FBG < 4.83 (mmol/L)	-14.01 (-24.97, -3.06)	0.0122	
FBG > 4.83 (mmol/L)	2.32 (1.30, 3.34)	<0.0001	
SUA < 420 μmol/L			<0.0001
FBG < 5.61 (mmol/L)	-1.45 (-2.67, -0.24)	0.0190	
FBG > 5.61 (mmol/L)	3.20 (2.49, 3.90)	<0.0001	
BMI < 25 Kg/m^2^			0.002
FBG < 5.22 (mmol/L)	-2.30 (-4.84, 0.24)	0.0763	
FBG > 5.22 (mmol/L)	2.66 (1.40, 3.93)	<0.0001	
BMI ≥25 Kg/m^2^			<0.001
FBG < 5.61 (mmol/L)	-2.09 (-3.71, -0.47)	0.0115	
FBG > 5.61 (mmol/L)	2.84 (2.07, 3.61)	<0.0001	
Non-hypertension #			<0.001
FBG < 6.00 (mmol/L)	-0.34 (-1.27, 0.60)	0.4798	
FBG > 6.00 (mmol/L)	5.02 (3.96, 6.08)	<0.0001	
Hypertension #			0.003
FBG < 5.44 (mmol/L)	-3.64 (-6.91, -0.37)	0.0294	
FBG > 5.44 (mmol/L)	1.99 (0.97, 3.01)	0.0001	
Non-diabetes *			<0.001
FBG < 5.72 (mmol/L)	-1.38 (-2.45, -0.30)	0.0119	
FBG > 5.72 (mmol/L)	4.28 (3.61, 4.95)	<0.0001	
Diabetes *			0.152
FBG < 5.11 (mmol/L)	-7.13 (-18.42, 4.16)	0.2160	
FBG > 5.11 (mmol/L)	1.23 (0.40, 2.07)	<0.0001	
Non-CVD			<0.001
FBG < 4.96 (mmol/L)	-1.22 (-1.80, -0.64)	<0.0001	
FBG > 4.96 (mmol/L)	0.63 (0.50, 0.76)	<0.0001	
CVD			0.013
FBG < 5.62 (mmol/L)	-1.30 (-2.54, -0.06)	0.0394	
FBG > 5.62 (mmol/L)	0.55 (0.07, 1.02)	<0.0001	
eGFR < 60 mL/min/1.73m^2^			0.165
FBG < 5.88 (mmol/L)	-0.99 (-7.62, 5.63)	0.7690	
FBG > 5.88 (mmol/L)	4.98 (1.52, 14.49)	0.0049	
eGFR ≥ 60 mL/min/1.73m^2^			0.020
FBG < 5.46 (mmol/L)	-2.13 (-3.48, -0.79)	0.0019	
FBG > 5.46 (mmol/L)	2.55 (1.91, 3.18)	<0.0001	
Non-smoking			<0.001
FBG < 5.22 (mmol/L)	-3.25 (-5.69, -0.80)	0.0092	
FBG > 5.22 (mmol/L)	2.14 (0.79, 3.49)	0.0019	
Smoking			<0.001
FBG < 5.72 (mmol/L)	-1.92 (-3.54, -0.29)	0.0208	
FBG > 5.72 (mmol/L)	3.89 (1.75, 6.03)	0.0004	
Drinking			<0.001
FBG < 5.53 (mmol/L)	-2.31 (-3.71, -0.91)	0.0012	
FBG > 5.53 (mmol/L)	2.72 (2.02, 3.43)	<0.0001	

FBG, fasting blood glucose; HbA1C, hemoglobin A1c; SUA, serum uric acid; BMI, body mass index; CVD, cardiovascular disease; UACR, urinary albumin-to-creatinine ratio.

Adjusted for gender, age, race, poverty income ratio, education level, creatinine; serum uric acid; blood urea nitrogen; triglyceride; high-density lipoprotein; low-density lipoprotein; estimated glomerular filtration rate; body mass index; smoking; drinking; diabetes; hypertension; cardiovascular disease; glucose-lowering therapy, antihypertension therapy, hypolipemic therapy. The above subgroups were adjusted for the variables, except for themselves.

#These subgroups were adjusted for the variables, except for themselves and antihypertension therapy.

*These subgroups were adjusted for the variables, except for themselves and glucose-lowering therapy.

## 4 Discussion

The present investigation conducted a comprehensive cross-sectional analysis to elucidate the correlation between FBG and UACR. To the best of our knowledge, this is the pioneering study to explore this particular relationship. Our findings indicate a significant association between elevated FBG levels and increased UACR. Furthermore, a U-shaped nonlinear pattern was observed in the relationship between FBG and UACR. Notably, we identified the inflection point of FBG. Specifically, when FBG levels were below 5.44 mmol/L, elevated FBG was linked with decreased UACR. Conversely, when FBG levels exceeded 5.44 mmol/L, a positive correlation between FBG and UACR was observed. These findings hold substantial implications in terms of understanding the relationship between FBG and UACR, particularly in cases of diabetes and its complications.

Diabetes mellitus is a cluster of metabolic disorders characterized by elevated fasting and postprandial blood glucose levels, which may result from insufficient insulin production or insulin resistance. Diabetes has demonstrated a progressive escalation in recent years, and it is projected to affect approximately 592 million people worldwide by 2035 (24). Diabetes-related complications, including diabetic nephropathy, retinopathy, neuropathy, cardiovascular complications, and hepatic fibrosis, can significantly impact the prognosis of individuals with diabetes (25). Maintaining stable blood glucose levels is of utmost importance, as it promotes effective management of blood glucose, which, in turn, aids in weight control and mitigates cardiovascular events (26, 27).

Albuminuria has been found to have detrimental effects, including an increased risk of recurrent venous thromboembolism (5%) (28), higher rates of hospitalization in the elderly (29), and an elevated risk of adverse cardiovascular events and mortality (30, 31). The presence of chronic hyperglycemia further amplifies the likelihood of developing albuminuria (32). Specifically, one mmol rise in mean FBG is associated with a 27% increase in proteinuria (33), ultimately culminating in the development of diabetic nephropathy and end-stage renal disease (34). Furthermore, it has been observed that inadequate regulation of glycemic levels in individuals with Young maturity-onset diabetes of the young type patients results in a significant 2-3-fold rise in the likelihood of developing microalbuminuria and retinopathy (35). This finding underscores the substantial impact of blood glucose on the occurrence of albuminuria.

A study conducted in Shanghai, China (36) revealed that the incidence of albuminuria was 28.9%, 28.1%, and 12.9% among individuals diagnosed with diabetes, individuals with undiagnosed diabetes, and individuals with pre-diabetes, respectively. Additionally, even among participants with normal blood glucose levels, the incidence of albuminuria was found to be 8.7%. Another study reached a similar conclusion, suggesting that poor glycemic control increases the likelihood of albuminuria (37). However, a study conducted in South Asia did not find a statistically significant association between decreased FBG levels and decreased UACR (14). In addition, an increase in albuminuria was observed in aged mice. However, blood glucose was indeed significantly reduced (38). The U-shaped association observed between FBG and UACR in our study provides a potential explanation for the conflicting findings regarding the relationship between FBG and UACR.

Numerous studies have elucidated the underlying mechanism through which increased FBG levels contribute to the occurrence of proteinuria, such as alterations in the glomerular filtration barrier (39, 40) and injury to podocytes (41). Conversely, limited research has been conducted on the mechanisms responsible for proteinuria within the normal range of FBG. However, the subsequent studies discussed herein may provide valuable insights into this matter. A study conducted by Kyung Chul Song (42) yielded a noteworthy finding: prolonged variations in glycemic control play a role in the emergence of microalbuminuria among individuals with type 1 diabetes, and mitigating glycemic fluctuations may potentially ameliorate diabetic microvascular complications (43). We propose that the manifestation of proteinuria in this cohort may be attributed to the presence of glucose fluctuations. Nevertheless, additional research is imperative to validate this hypothesis. In addition, it is plausible that this cohort of individuals exhibited insulin resistance despite having normal blood glucose levels (44). Given that the extent of insulin resistance is closely associated with the likelihood of developing CKD (45), it follows that insulin resistance contributes to elevated urinary albumin excretion (46, 47).

The risk factors for microalbuminuria include aging, female gender, obesity, elevated SUA levels, hypertension, diabetes, smoking, and alcohol consumption (48–51). Consequently, we investigated the association between FBG and UACR in various U.S. populations, considering factors such as gender, age, BMI, SUA, eGFR levels, past medical history, and smoking and alcohol habits. Our study revealed a U-shaped relationship between FBG and UACR in diverse populations, except for those with SUA levels ≥ 420 μmmol/L and individuals who do not consume alcohol, suggesting the relative stability of our findings.

It is crucial to acknowledge the limitations inherent in our study. Firstly, the cross-sectional design restricts our ability to establish a causal relationship between FBG and UACR, thereby underscoring the necessity for future investigations employing randomized controlled trials. Secondly, to ensure data integrity, we have excluded a substantial proportion of participants, potentially compromising the generalizability of our findings to the broader U.S. population. Moreover, the applicability of our study’s results to populations in different countries or regions requires further exploration. Moreover, while UACR may serve as a valuable indicator for evaluating albuminuria (52), it is critical to acknowledge that quantifying urine protein over 24 hours remains the gold standard. Further investigation is required to determine if comparable outcomes can be observed between FBG and 24-hour urine protein measurements. Ultimately, our study found no evidence of a U-shaped relationship between FBG and UACR in populations with SUA levels ≥420 μmol/L and those who do not drink alcohol. Nevertheless, the precise influence of hyperuricemia and non-alcohol consumption on FBG and UACR necessitates further investigation. These findings invite further inquiry into the interactions between these factors and their impact on metabolic health.

## 5 Conclusion

Our study has revealed a U-shaped non-linear association between FBG and UACR among the general population in the U.S. We found that when FBG levels surpassed a particular threshold, UACR levels increased with FBG levels. These findings may have significant implications for the management of diabetes and associated complications.

## Data availability statement

Publicly available datasets were analyzed in this study. This data can be found here: https://www.cdc.gov/nchs/nhanes/index.htm.

## Author contributions

JS: Writing – original draft, Software, Investigation, Conceptualization. PW: Writing – review & editing, Supervision, Methodology, Data curation. HL: Writing – review & editing.
