# Supplementary material for: U-shaped relationship between fasting blood glucose and urinary albumin-to-creatinine ratio in the general United States population
Source: Front Endocrinol (Lausanne). 2024 Mar 15;15:1334949. doi: 10.3389/fendo.2024.1334949 (PMC10978799; doi:10.3389/fendo.2024.1334949)
Supplement: Supplementary file 1 [file Table_1.docx]

Supplement table 1 Univariate analysis of variables associated with UACR

|  | UACR |
| --- | --- |
| Age (years) | 1.13 (0.89, 1.37) <0.0001 |
| Sex [N (%)] |  |
| Female | 0 |
| Male | 4.23 (-4.19, 12.65) 0.3252 |
| Race [N (%)] |  |
| Non-Hispanic white | 0 |
| Non-Hispanic black | 11.62 (-2.35, 25.59) 0.1029 |
| Mexican American | -21.51 (-33.55, -9.47) 0.0005 |
| Other | -6.50 (-20.91, 7.90) 0.3762 |
| Education level [N (%)] |  |
| Junior high school education or below | 0 |
| High school education | -15.68 (-27.94, -3.41) 0.0122 |
| College education or above | -28.51 (-38.86, -18.16) <0.0001 |
| PIR (%) | -8.28 (-10.87, -5.68) <0.0001 |
| FBG (mmol/L) | 23.93 (21.69, 26.17) <0.0001 |
| HbA1C (%) | 48.85 (44.87, 52.83) <0.0001 |
| Cr (mmol/L) | 2.88 (2.76, 3.00) <0.0001 |
| SUA (μmol/L) | 0.26 (0.21, 0.31) <0.0001 |
| BUN (mmol/L) | 29.64 (27.63, 31.66) <0.0001 |
| TG (mmol/L) | 20.37 (14.81, 25.93) <0.0001 |
| TC (mmol/L) | 4.07 (0.06, 8.08) 0.0466 |
| HDL (mmol/L) | -7.67 (-17.93, 2.59) 0.1429 |
| LDL (mmol/L) | 0.54 (-4.03, 5.11) 0.8169 |
| eGFR (mL/min/1.73m^2^) | -2.07 (-2.25, -1.89) <0.0001 |
| BMI (Kg/m^2^) | 1.76 (1.15, 2.36) <0.0001 |
| Smoking [N (%)] |  |
| No | 0 |
| Yes | 2.71 (-5.73, 11.16) 0.5293 |
| Drinking [N (%)] |  |
| No | 0 |
| Yes | -23.14 (-35.61, -10.67) 0.0003 |
| Diabetes [N (%)] |  |
| No | 0 |
| Yes | 142.63 (130.06, 155.19) <0.0001 |
| Hypertension [N (%)] |  |
| No | 0 |
| Yes | 61.55 (53.06, 70.05) <0.0001 |
| CVD [N (%)] |  |
| No | 0 |
| Yes | 75.32 (61.74, 88.90) <0.0001 |
| CKD [N (%)] |  |
| No | 0 |
| Yes | 197.47 (186.69, 208.26) <0.0001 |
| Glucose-lowering therapy |  |
| No | 0 |
| Yes | 74.80 (46.65, 102.96) <0.0001 |
| Antihypertension therapy |  |
| No | 0 |
| Yes | 60.11 (49.38, 70.84) <0.0001 |
| Hypolipemic therapy |  |
| No | 0 |
| Yes | -24.56 (-49.80, 0.69) 0.0566 |

PIR: poverty income ratio; FBG: fasting blood glucose; HbA1C: hemoglobin A1c; Cr: creatinine; SUA: serum uric acid; BUN: blood urea nitrogen; TG: triglyceride; TC: total cholesterol; HDL: high-density lipoprotein; LDL: low-density lipoprotein; UACR: urinary albumin-to-creatinine ratio; eGFR: estimated glomerular filtration rate; BMI: body mass index; CVD: cardiovascular disease; CKD: chronic kidney disease.
